# Relationship between covid-pandemic anxiety and sleep disorder with menstrual disorders among female medical workers

**DOI:** 10.1186/s12905-023-02314-2

**Published:** 2023-04-28

**Authors:** Wenxi Sun, Liping Xia, Caifang Ji, Qianqian Wei, Jun Zhang, Sidi He, Xuqin Wang, Xueqin Shen, Xiaobin Zhang, Chuanwei Li

**Affiliations:** 1 Psychiatry department of Suzhou Guangji Hospital, Affiliated Guangji Hospital of Soochow University, Suzhou, 215137 Jiangsu Province China; 2 The Affiliated Jiangsu Shengze Hospital of Nanjing Medical University, Suzhou, 215100 Jiangsu Province China

**Keywords:** COVID-19, Menstrual cycle, Female, Medical workers, Cross-sectional survey, China

## Abstract

**Background:**

It has been more than 2 years since the 2019 novel coronavirus disease (COVID-19) pandemic destabilized the world, adversely affecting not only physical health, but also mental health. During this time, frontline medical workers were at a greater health risk, especially female medical workers. Changes or abnormalities in the menstrual cycle—an important indicator of women’s health—may jeopardize female reproductive functioning. Considering that emotional health and sleep status may be related to the menstrual cycle, this study aimed to investigate the association between menstrual cycle changes, anxiety, sleep dysfunction, and other factors among female medical workers during the COVID-19 pandemic.

**Methods:**

A cross-sectional survey was conducted by distributing online questionnaires to female medical workers in China from February to May 2022. The study included 160 women aged 18–45 years old. The questionnaires covered data related to the participants’ sociodemographic characteristics, medical and reproductive history, and lifestyle. The Rating Scale for Clinical Manifestations of Menopathy (SCMM), Self-Rating Anxiety Scale (SAS), and Sleep Dysfunction Rating Scale (SDRS) were utilized. Data were analyzed using chi-square tests, t-tests, and linear regression analysis.

**Results:**

A total of 160 female medical staff were randomly selected in this research, of whom seven scored less than 3 points, 85 scored 3–11 points, and 68 scored more than 11 points on the total score of the SCMM. Compared to pre-pandemic scores, scores of dizziness and tinnitus were significantly higher during the COVID-19 pandemic. Scores corresponding to the following clinical symptoms were also higher during the pandemic: Menopathy, including hypaphrodisia, dim complexion, abnormal urination, languidness, dim menstruation, thin menstruation, dysmenorrhea, and empty or saggy lower abdomen (p < 0.05). However, pre-pandemic scores of vaginal bleeding quantity were significantly higher than those found during the COVID-19 pandemic (p < 0.05). Scores of vaginal bleeding quantity were significantly lower in cabin hospitals than other types of hospitals, and a similar finding was observed for vaginal bleeding duration (all p < 0.05). Moreover, the findings of the univariable and multivariable linear regression analysis revealed a link between consistent exercise, the underlying illness, the SDRS score, the SAS score, and the total score of SCMM (p < 0.05).

**Conclusions:**

In this study, we found that menstruation in female medical workers was affected by the COVID-19 pandemic. Furthermore, regular exercise and good physical condition were protective factors, while anxiety and insomnia were risk factors for menstrual abnormalities.

## Introduction

The normal menstrual cycle is the result of a highly coordinated hypothalamic-pituitary-ovarian (HPO) axis with complex hormonal feedback loops that lead to the formation of a dominant follicle, ovulation, and in the absence of fertilization, shedding of the endometrial lining at regular intervals [1]. The menstrual cycle is multifaceted and characterized by the premenstrual phase, during which women may experience somatic complaints (e.g., breast tenderness and abdominal bloating) and psychological distress (e.g., tension, irritability, dysphoria) [2], in addition to the menses, causing symptoms such as heavy bleeding and bothersome cramping [3]. A healthy menstrual cycle is an important indicator of a woman’s well-being [4]. Menstrual problems, however, are exceedingly prevalent and crippling [5]. A large socioeconomic burden is placed on women, their families, the healthcare system, and society as a whole as a result of problematic menstruation, which can also cause anemia [6], and a considerable detrimental effect on quality of life is observed [7, 8]. Traditional Chinese medicine views problematic menstruation as a disease that is characterized by an abnormal menstruation cycle, period, volume, color, and quality, accompanied by a menstruation cycle, or obvious symptoms before and after menstruation break [9]. Gynecological clinics are frequently attended for treatment of this condition. Previous studies have identified several correlates, including hormonal irregularities, vitamin deficiencies, prostaglandin and neurotransmitter dysregulation, psychosocial factors [10], and atypical responses to changes during the menstrual cycle [11]. Indeed, in yet another study conducted by Saunders and Hawton [12], women may be at risk of a greater incidence of completed suicides and suicide attempts in the menstrual phase. In addition, according to the findings of one prospective cohort research, irregular and prolonged menstrual periods are linked to a higher risk of premature death [4]. Therefore, the elucidation of related or preventable factors of menstrual cycle irregularity is essential for women’s health.

Over the past 2 years, our lives have been profoundly impacted by COVID-19 and the limitations that it imposed on daily activities. As of October 12, 2022, a total of 619,770,633 confirmed cases of COVID-19 were reported to the World Health Organization, including 6,539,058 deaths [13]. COVID-19 is not only capable of causing respiratory symptoms; it may also cause harm to other bodily systems, such as the nervous and immune systems [14, 15] or liver [16], and in a few rare cases, it can damage the reproductive system [17]. Psychological factors such as stress, anxiety, and depression can cause menstrual irregularities and amenorrhea in women [18]. In September 2020, 1,031 women of reproductive age responded anonymously to a digital survey conducted by Phelan et al. as part of an observational study of women’s reproductive health throughout the pandemic. The researchers discovered significant increases in depressive symptoms, poor appetite, inability to focus, anxiety, restlessness, and loneliness [19]. The COVID-19 pandemic has caused psychological distress, especially among female medical workers. There is an important public health need for accurate scientific investigation of these phenomena.

Nevertheless, to date, the majority of COVID-19 studies [20–25] concerning the menstrual cycle have focused on patients during the COVID-19 pandemic, while little research has been carried out to examine menstrual health for frontline medical workers. However, Huang and Zhao [26] found that healthcare workers were more at risk of mental health problems. Thus, this study aimed to systematically analyze the epidemiological and clinical characteristics of medical workers, and to identify any effects that the COVID-19 pandemic has had on the menstrual cycle. A cross sectional research was conducted using clinical data and data related to menstrual changes, COVID-19 pandemic-related anxiety, sleep dysfunction, and other factors among medical workers.

## Methods

### Participants

We conducted a cross-sectional study by administrating online questionnaires to female medical workers in China from February to May 2022. This survey was carried out in cabin hospitals, designated hospitals, and sampling sites, and involved healthcare workers including physicians and nurses.

To calculate the sample size, the following formula we used:

N = u*P*(1-P)/ δ ^2^, where u = 1.96 at a 95% confidence interval. P indicates a prevalence rate of 20%, taken from the previous study [27], and δ expresses an expected error rate of 5%. The required sample size was N = 1.96*0.2*(1-0.2)/0.05^2^ =125.

The random sampling process can be described as follows:

Using a sequential technique, the sample unit was drawn by choosing any digital number listed in the random number table. A digital number was selected from within the range. Numbers beyond the range of the selected digital number were not selected, repeated digital no longer selected, until the predetermined sample capacity was reached.

In this study, the medical workers were requested to join a WeChat group, at which time the purpose, nature, and administration procedures of the research were explained to them. Each participant provided informed consent and sent the link to our questionnaire. Participants inside the group accepted the invitation to complete the questionnaire anonymously.

During the recruitment phase, a total of 1801 medical workers were fighting the COVID-19 pandemic, 1170 of whom were female. We employed a simple random sampling method to select 212 medical workers from among this group and sent survey invitations. A total of 179 medical workers who were contacted, agreed to take part, and 33 did not reply to the invitation. In addition, 19 individuals were excluded, 13 failed to meet the inclusion criteria, and six provided only partial responses. The final analysis included 160 individuals after ineligible participants were eliminated.

#### The inclusion criteria

Female medical workers aged 18**–**45 years who had been involved in the fight against the COVID-19 pandemic in China were included in the study. The study group represented a special group of medical workers who were directly involved in the treatment of COVID-19-infected patients in the outbreak area. The participants were divided into seven groups, each of which consisted of 6–10 doctors and 15–25 nurses, and each group was assigned to 200**–**300 patients. The participants worked 4 h per day, in turn, day and night, until the outbreak is under control.

#### The exclusion criteria

Women who were pregnant, postpartum or lactating, taking hormonal or other types of medication (e.g., sex hormones, vaginal administration, anticoagulants, etc.) that could affect their menstrual patterns, or who had intrauterine devices, bleeding disorders, thyroid disorders, hyperprolactinemia, chronic renal failure, or cancer, had undergone hysterectomy and/or oophorectomy, approaching menopause, had gynecological disorders that affected their menstrual cycles, or were diagnosed with sleep disorders or a serious psychiatric disorder were not included in the study.

### Measures

Participants completed the questionnaire which included four sub-instruments as follows: (1) sociodemographic characteristics, medical and reproductive history, and lifestyle information; (2) the Rating Scale for Clinical Manifestation of Menopathy (SCMM); (3) Self-Rating Anxiety Scale (SAS); and (4) Sleep Dysfunction Rating Scale (SDRS).

#### SCMM

The SCMM mainly evaluates the participants’ menstrual status. According to the diagnostic criteria of menopathy in traditional Chinese medicine, the scale was developed by drawing on the “Guiding Principles for Clinical Research of New Chinese Medicines.” It contains 15 test items and is a four-point Likert scale. The SCMM consists of two subscales (Cardinal Symptoms and secondary symptoms) [28]. Cardinal Symptoms contain the first six items and secondary symptoms are assessed by the last nine items. Higher scores indicate greater severity of menopathy. This scale has a wide range of applications [29, 30].

#### SAS

The SAS was compiled by Zung (1971) [31] and contains 20 test items and a four-point Likert scale. It mainly evaluates the participants’ anxiety status measured with a one-week interval. It is suitable for all occupations, cultures, ages, and adults with anxiety symptoms, and has a wide range of applications [32, 33]. Higher scores indicate more serious levels of anxiety.

#### Sleep dysfunction rating scale, SDRS

The scale was developed by Xiao et al. as an instrument to measure sleep dysfunction. According to the classification and diagnostic criteria of mental disorders in China, the scale draws on the experience of other sleep disorder rating scales. The SDRS consists of 10 items, and the scores for each item range from 0 ~ 4 points. Each item has evaluation guidelines and scoring criteria. Higher scores indicate greater severity of sleep dysfunction. The validity and reliability of this scale for use in those suffering from insomnia was established in a previous study [34].

We first assessed participants’ menstrual cycle changes using the SCCM. We then assessed mood and sleep status using the SAS and SDRS, and combined the sociodemographic characteristics to analyze the relationship between menstrual cycles and mood, sleep, vaccination and viral infection during post-epidemic period.

### Statistical analysis

Data analysis was performed using Windows-compatible SPSS version 25 software. For categorical variables, descriptive statistics were reported as frequencies (percentages), and continuous data presented as mean ± standard deviation. Tests for sphericity and homogeneity of variance were run on the data to compare the continuous variables. Using Student’s t-test, continuous variables that adhered to the assumptions of normality were examined. In order to identify the determinants of “the total score of the SCMM,“ variables that were shown to be statistically significant by the univariate analysis were also incorporated in a multivariate logistic regression model. P values of 0.05 were regarded as statistically significant for all statistical tests.

### Ethics

This study was approved by the ethics committee of the affiliated Guangji Hospital of Soochow University, and all participants provided online informed consent. The research was conducted in accordance with the Declaration of Helsinki and its later amendments or comparable ethical standards, and all procedures were completed. Participants were informed about the purposes of the study, its nature, and the procedures involved. They were assured that all information given by them would be stored confidentially and anonymously.

## Results

Menstrual cycle characteristics. Frequency, short: shorter than 25 days; normal: between 25 and 35 days; long: longer than 35 days. Duration, short: shorter than 3 days; normal: between 3 and 7 days; long: longer than 7 days. Quantity, heavy: more than 80 ml; normal between 20 and 80 ml; light: less than 20 ml.

A total of 160 women were included in the study, of whom seven scored less than 3 points, 85 scored 3**–**11 points, and 68 scored more than 11 points on the total score of clinical symptoms of menopathy. The baseline sociodemographic and clinical characteristics of the group are detailed in Table 1.

**Table 1:** Characteristics of female medical workers during the COVID-19 pandemic

Demographics		
Age, years (mean ± SD)	31.84 ± 5.97	
Engagement time, seconds (mean ± SD)	516.59 ± 254.58	
Region(n[%])		
Nanjing, jiangsu province	8	5.000%
Changzhou, jiangsu province	1	0.625%
Suzhou, jiangsu province	54	33.750%
Zhenjiang, jiangsu province	48	30.000%
Nantong, jiangsu province	1	0.625%
Hangzhou, zhejiang province	46	28.750%
Shanghai	2	1.250%
Medical profession (n[%])		
Nurse	151	94.375%
Doctor	9	5.625%
Rank(n[%])		
Primary	82	51.250%
Middle	64	40.000%
Senior	14	8.750%
Workplace(n[%])		
Cabin	140	87.500%
Hospital	9	5.625%
sampling site	11	6.875%
Basic disease(n[%])		
Yes	16	10.000%
No	144	90.000%
Medication(n[%])		
Yes	18	11.250%
No	142	88.750%
Regular exercise(n[%])		
Yes	65	40.625%
No	95	59.375%
Marital status(n[%])		
Single	49	30.625%
Married	29	18.125%
With child	82	51.250%

Table 2 shows the average score of clinical symptoms of menopathy before and during the COVID-19 pandemic. A significant difference between groups was observed with regards to clinical symptoms of menopathy, including vaginal bleeding quantity, dizziness and tinnitus, hypaphrodisia, dim complexion, urination disorder, languidness, dim menstruation, thin menstruation, dysmenorrhea, and empty or saggy lower abdomen (p < 0.05). Scores of dizziness and tinnitus during the COVID-19 pandemic were significantly higher than pre-pandemic scores (t=-3.831, p < 0.001), and scores of its following clinical symptoms of the menstrual disease: hypaphrodisia (t=-2.956, p < 0.05), dim complexion (t=-4.800, p < 0.001), urination disorder (t=-4.092, p < 0.001), languidness(t=-2.851, p < 0.05), dim menstruation (t=-3.668, p < 0.001), thin menstruation(t=-4.232, p < 0.001), dysmenorrhea(t=-2.509, p < 0.05), and empty or saggy lower abdomen (t=-4.099, p < 0.001). However, scores of vaginal bleeding quantity before the pandemic were significantly higher than during the pandemic (t = 3.514, p = 0.001). There was no significant difference between groups regarding clinical symptoms of menopathy including vaginal bleeding duration, vaginal bleeding period, lumbosacral ache, abnormal menstruation, and empty or saggy lower abdomen (p > 0.05).

**Table 2:** Comparison of clinical symptoms of menopathy among female medical workers before and during the COVID-19 pandemic

	Before pandemic(N = 160)	During pandemic(N = 160)	Statistic	P
Cardinal Symptom, points (mean ± SD)				
Vaginal bleeding quantity	1.210 ± 0.891	1.030 ± 0.893	t = 3.514	0.001
Vaginal bleeding duration	0.360 ± 0.619	0.370 ± 0.660	t=-0.124	0.902
Vaginal bleeding period	0.610 ± 0.985	0.730 ± 1.009	t=-1.906	0.058
Lumbosacral ache	0.980 ± 0.682	1.010 ± 0.828	t=-0.639	0.524
Dizziness and tinnitus	0.400 ± 0.563	0.560 ± 0.750	t=-3.831	0.000
Hypaphrodisia	0.450 ± 0.791	0.580 ± 0.873	t=-2.956	0.004
Secondary symptom, points (mean ± SD)				
Dim complexion	0.740 ± 0.820	0.990 ± 0.928	t=-4.800	0.000
Urination disorder	0.200 ± 0.473	0.360 ± 0.676	t=-4.092	0.000
Languidness	0.880 ± 0.707	1.030 ± 0.793	t=-2.851	0.005
Dim menstruation	0.540 ± 0.726	0.740 ± 0.835	t=-3.668	0.000
Thin menstruation	0.330 ± 0.610	0.550 ± 0.751	t=-4.232	0.000
Dysmenorrhea	0.890 ± 0.727	0.990 ± 0.832	t=-2.509	0.013
Abnormal menstruation	1.170 ± 1.161	1.270 ± 1.201	t=-1.696	0.092
Cold limb and cold intolerance	0.910 ± 0.893	0.990 ± 0.942	t=-1.872	0.063
Empty or saggy lower abdomen	1.020 ± 0.713	1.200 ± 0.830	t=-4.099	0.000

Table 3 shows the average score of clinical symptoms of menopathy among participants in different workplace environments. A significant difference between the groups was observed with regards to vaginal bleeding quantity and vaginal bleeding duration (all p < 0.05). Scores of vaginal bleeding quantity among female medical workers in the cabin hospitals were significantly lower than those of workers in other hospitals (t=-3.422, p < 0.001), and a similar finding was observed in the case of vaginal bleeding duration (t=-2.058, p < 0.05). Although all other menstrual symptom scores were lower among female medical workers in cabin hospitals than among those working in other workplace environments, none of the differences were significant.

**Table 3:** Comparison of clinical symptoms of menopathy among female medical workers in different workplace environments

	Cabin hospital (N = 140)	Sampling site/hospital(N = 20)	Statistic	P
Cardinal Symptom, points (mean ± SD)				
Vaginal bleeding quantity	0.940 ± 0.863	1.650 ± 0.875	t=-3.422	0.001
Vaginal bleeding duration	0.330 ± 0.651	0.650 ± 0.671	t=-2.058	0.041
Vaginal bleeding period	0.610 ± 0.985	0.730 ± 1.009	t=-0.117	0.908
Lumbosacral ache	0.720 ± 1.011	0.750 ± 1.020	t=-0.252	0.801
Dizziness and tinnitus	1.000 ± 0.814	1.050 ± 0.945	t=-0.828	0.416
Hypaphrodisia	0.540 ± 0.743	0.700 ± 1.031	t=-0.684	0.495
Secondary symptom, points (mean ± SD)				
Dim complexion	0.980 ± 0.933	1.100 ± 0.912	t=-0.546	0.586
Urination disorder	0.340 ± 0.642	0.500 ± 0.889	t=-0.798	0.434
Languidness	1.010 ± 0.786	1.100 ± 0.852	t=-0.451	0.652
Dim menstruation	0.690 ± 0.821	1.050 ± 0.835	t=-1.801	0.074
Thin menstruation	0.540 ± 0.772	0.600 ± 0.598	t=-0.318	0.751
Dysmenorrhea	0.960 ± 0.817	1.150 ± 0.933	t=-0.934	0.352
Abnormal menstruation	1.210 ± 1.186	1.650 ± 1.268	t=-1.524	0.130
Cold limb and cold intolerance	0.950 ± 0.884	1.300 ± 1.261	t=-1.200	0.243
Empty or saggy lower abdomen	1.180 ± 0.807	1.350 ± 0.988	t=-0.863	0.389

Univariable and multivariable linear regression analysis was carried out to assess factors associated with menstrual cycles (Table 4). The univariable linear regression analysis produced the following results: regular exercise (p < 0.001, odds ratio [OR]=-4.411, 95% confidence interval [CI] -6.533–-2.288); basic disease (p < 0.05, OR=-3.813, 95%CI -7.419–-0.206); SDRS score (p < 0.001, OR=-0.469, 95%CI 0.345–0.593); and SAS score (p < 0.001, OR = 0.118, 95%CI 0.067–0.169). The multivariable linear regression analysis was conducted using variables that were found to be significant in the univariate model. The multivariate model was significant: (χ2 (5) = 23.078, p < 0.001), and revealed that regular exercise (p < 0.001, OR = -3.592, 95%Cl -5.390–-1.793), basic disease (p < 0.05, OR =-3.084, 95%Cl -6.013–-0.154), the SDRS score (p < 0.001, OR = 0.378, 95%Cl 0.254–0.503), and SAS score (p < 0.05, OR = 0.063, 95%Cl 0.016–0.110) were significant predictors of the total SCMM score.

**Table 4:** Results of the univariate and multivariate linear regression analysis of the total SCMM score

	Univariate		Multivarite	
	Odd ratio(95%CI)	P value	Odd ratio(95%CI)	P value
Age	0.075(-0.109,0.259)	0.423		
Marital status	0.268(-0.963,1.500)	0.677		
Take medication	-1.574(-5.035,1.888)	0.371		
Regular exercise	-4.411(-6.533,-2.288)	0.000	-3.592(-5.390,-1.793)	0.000
Basic disease	-3.813(-7.419,-0.206)	0.038	-3.084(-6.013,-0.154)	0.039
SDRS	0.469(0.345,0.593)	0.000	0.378(0.254,0.503)	0.000
SAS	0.118(0.067,0.169)	0.000	0.063(0.016,0.110)	0.009

## Discussion

In this survey study of female medical workers aged between 18 and 45 years old during the COVID-19 pandemic, it was observed that the vaginal bleeding quantity of the participants decreased during the pandemic, while other clinical symptoms of menopathy (i.e., dizziness and tinnitus, hypaphrodisia, dim complexion, urination disorder, languidness, dim menstruation, thin menstruation, dysmenorrhea, and empty or saggy lower abdomen) increased compared to the pre-pandemic period. The scores of vaginal bleeding quantity were significantly lower among female medical workers in cabin hospitals than the scores of those working in other clinical environments, and a similar finding was observed in the case of vaginal bleeding duration. The present study found that physical health was negatively associated with menstrual cycle problems, which indicated that better physical health was associated with fewer menstrual-related problems. In addition, it was observed that the SAS and SDRS scores predicted the total score of clinical symptoms of menopathy, and this result was statistically significant.

It is widely accepted that the majority of women around the world experience menstruation, and gynecologists are frequently consulted to address menstrual problems. Abnormal menstrual cycles are a common source of concern, particularly in relation to fertility and increased risk of premature death [35]. The COVID-19 outbreak in 2019 spread quickly around the world. Most cases of COVID-19 were mild and did not require medical assistance; however, 15–20% of those infected were hospitalized, and 5% required mechanical ventilation [36]. In the post-COVID-19 period, an upsurge has been observed in menstrual abnormalities such as prolonged cycles and decreased menstrual volume [19]. Women who occasionally missed a period before the pandemic reported an increase in missed periods during the pandemic. Similar experiences were reported in the current investigation, namely significant changes in menstrual-related characteristics (vaginal bleeding quantity, dizziness, and tinnitus, hypaphrodisia, etc.) among female healthcare workers during the COVID-19 pandemic. It has long been demonstrated that external factors can adversely affect the female menstrual cycle [37–39]. According to previous research [39, 40], the hypothalamic–pituitary–adrenal (HPA) axis is sensitive to stressors (e.g. war, dilemmas, and anxiety), leading to changes in hormonal pulsation mechanisms, which in turn affect the female menstrual cycle. Therefore, we propose that aberrant alterations in the female menstrual cycle may occur as a result of changes in hormone levels caused by the stress of COVID-19. However, the effect of COVID-19 on the menstrual cycle remains largely unclear, and further research is still needed to determine the specific mechanism of action between the two. The current study found a strong correlation between menstrual cycle irregularity and the physical condition of the medical workers. Daily physical exercise has also been linked to increased insulin sensitivity and body weight maintenance, both of which can help to regulate the menstrual cycle [41]. In addition, there is growing evidence that healthy behaviors including alterations in alcohol use, food, and physical exercise might affect the menstrual cycle [42]. Lockdowns and social seclusion were designed to mitigate COVID-19, and were epidemic control techniques that limited movement and caused annoyance for everyone. As a result, normal, moderate physical activity can contribute to regular menstruation, although there is a need to remain mindful of the importance of self-protection during this current COVID-19 epidemic.

The National Health Commission declared that, by the end of 2020, there will be 3.4 million registered practicing physicians in China, and women are expected to account for about half of this figure. It is estimated that there will be 4.7 million female nurses on the register, with women accounting for 97%. The results of Xu Ming et al.’s research [43] on the reproductive health of female workers in seven provinces and cities in China found higher abnormal menstrual rates and gynecological physical examination rates among medical workers than among other occupational groups. Reproductive health issues among female medical workers are a major concern. The results of studies carried out by Thurston et al. [44] and Christiani et al. [45] suggested that work-related stress and dysmenorrhea are inversely connected. In other words, stressful work settings may adversely affect menstrual function, according to Wang et al. [46] who discovered that nurses are more likely to have protracted and monophasic periods (RR = 4.3 and 5.5, respectively). The results of this study found higher scores among female medical workers in designated hospitals or sampling points compared to those who worked in cabin hospitals. Moreover, significantly higher scores of vaginal bleeding quantity and vaginal bleeding duration were found among workers in designated hospitals than among those in cabin hospitals. During the current COVID-19 pandemic, cabin hospitals are typically used to accommodate asymptomatic infections or mild patients, whereas patients in designated hospitals are more seriously ill. In addition, medical workers at sampling points should the collection of throat swabs for suspected people and key screening groups. Therefore, compared to designated hospitals and sampling points, the workload of medical workers in cabin hospitals is light. This means that medical workers who are charged with heavy workloads are more likely to experience menstrual problems. This is consistent with the conclusions of the above study.

The results of the current study revealed that the COVID-19 pandemic had a significant negative impact on both the physical and mental health of a significant portion of the population, resulting in isolation, economic stress, anxiety, and fear of contracting the virus, as well as uncertainty about the future [47]. Typhoons [48], floods [49], and epidemics [50] are only a few examples of major life events that have been linked to psychological stress, according to earlier research [51]. The COVID-19 pandemic is no exception. Since the outbreak of COVID-19, a significant increase has been observed in reported acute mental health problems. Research has also shown that up to 84% of women reported that they experienced at least one mental health symptom, with low mood, anxiety, and insomnia being the most common [19]. Furthermore, it was discovered that the COVID-19 epidemic had increased levels of anxiety and tension, to the extent that these symptoms had an impact on the menstrual cycle of the women polled [52]. In our investigation, we found similar results. In addition, we discovered other evidence, such as the fact that nearly half of the women who participated in this study reported that they had trouble sleeping, and these women were more likely to experience changes in their menstrual cycle [19]. Although this study was only a cross-sectional investigation, our findings provide room for speculation that anxiety levels and sleep disturbances are predictive of menstrual disorders in female medical workers. Moreover, research has suggested that, compared to non-healthcare professionals, healthcare workers—especially female healthcare workers—experience more mental health issues, such anxiety and sadness [53–55]. The issues presented above can be further investigated in our upcoming study.

Efforts to curb the spread of COVID-19 have involved the implementation of measures including isolation and observation of suspected cases, and vaccination of susceptible populations. As a result, several vaccines were produced and rolled out for clinical use, such as Comirnaty (Pfizer BioNTech), Spikevax (Moderna), and Vaxzevria (AstraZeneca), among others. Frontline medical workers were prioritized for vaccination. Common adverse reactions in people after vaccination with the COVID-19 vaccine include clinical manifestations such as myalgia, pain in the vaccinated arm, fever and weakness [56]. Changes in the menstrual cycles of women of childbearing age after vaccination with the COVID-19 vaccine have also caused widespread concern among health professionals [57, 58]. However, such observations do not necessarily indicate a causal relationship [59], and clinical manifestations following vaccination are likely due to a COVID-19 infection. In gynecological clinical trials, an increasing number of women have reported transitory menstrual changes and irregular menstruation immediately after immunization [60], and all of these reported changes seemed to return to normal in the next cycle [61]. The above clinical manifestations may be further explained by the mechanisms associated with SARS-CoV-2 infection. Given that SARS-CoV-2 infection and COVID-19 may affect the hypothalamic-pituitary-ovarian-intrauterine axis, causing changes in the menstrual cycle such as decreased menstrual volume and prolonged menstrual cycles [62], this does not seem to be linked to COVID-19 severity. The reproductive system and SARS-CoV-2 infection may interact in a more precise manner. This may occur at the ovarian/endometrial level. Progesterone is primarily an anti-inflammatory hormone and is present in the ovaries [63]. During the premenstrual period, progesterone levels drop dramatically, leading to an influx of inflammatory cells into the local endometrial environment, which eventually leads to the shedding of functional endometrium during menstruation [64]. Therefore, considering that COVID-19 vaccination induces an immune response, the subsequent inflammation can transiently interfere with ovarian hormone production for one or two cycles, leading to abnormal menstrual bleeding [65]. Nonetheless, further clinical evidence is needed to support the aforementioned viewpoint, and we cannot rule out the possibility of long-term consequences following COVID-19 immunization.

### Limitations of the study

This study had several limitations. First, a relatively small number of female medical workers were enrolled. Second, the questionnaire responses could have been affected by recollection bias. However, a study by Sampson and Prescott found that self-rating is typically the most practical tool available, and it may be used to evaluate both premenstrual and menstrual symptoms [62]. Third, no information was available with respect to the mental health of the medical workers prior to the COVID-19 pandemic. Finally, the causal relationship between the variables cannot be established using a cross-sectional design. The causal connection between menstrual cycle irregularity, sleep characteristics, and mental health calls for further investigation. Despite these shortcomings, we believe that the use of digital questionnaires for the purposes of conducting research on reproductive health among medical professionals during the COVID-19 pandemic is reliabled and validited.

### The strength of the study

The key strength of this research is that it is the first study by Traditional Chinese Medicine to examine the relationship between the COVID-19 pandemic and the menstrual cycle. The participants included female frontline medical workers, and physical health, anxiety, sleep dysfunction, and menstruation symptoms were evaluated by administering validated questionnaires. Another strength of this study is that the majority of the participants used a smartphone application to record their menstrual cycle patterns, meaning that menstrual cycle data are likely to be unbiased.

## Conclusions

In conclusion, the results indicated an association between irregular exercise, basic disease, anxiety, sleep dysfunction, and increased levels of menstrual cycle irregularity among female medical workers during the COVID-19 pandemic. Therefore, more detailed investigations are needed to assess the complicated relationships between physical health, anxiety, sleep, and the menstrual cycle. This will facilitate the development of strategies, which can help improve the physical and emotional health of women suffering from menstrual disturbances during the COVID-19 pandemic.

## Data Availability

The datasets generated and/or analyzed in the current study are not publicly available due to be further explored, but are available from the corresponding authors upon reasonable request.

## Abbreviations

COVID-19: Coronavirus disease 2019
SCMM: Rating Scale for Clinical Manifestation of Menopathy
SAS: Self-Rating Anxiety Scale
SDRS: Sleep Dysfunction Rating Scale
